# Liver Steatosis: Better Predictor of CKD in MAFLD Than Liver Fibrosis as Determined by Transient Elastography With Controlled Attenuation Parameter

**DOI:** 10.3389/fmed.2021.788881

**Published:** 2022-01-13

**Authors:** Luciana Marc, Adelina Mihaescu, Raluca Lupusoru, Iulia Grosu, Florica Gadalean, Flaviu Bob, Lazar Chisavu, Nicu Olariu, Vlad Tucicovschi, Bogdan Timar, Ioan Sporea, Romulus Timar, Adalbert Schiller

**Affiliations:** ^1^Department of Internal Medicine II – Division of Nephrology, “Victor Babes” University of Medicine and Pharmacy, Timişoara, Romania; ^2^Centre for Molecular Research in Nephrology and Vascular Disease, Faculty of Medicine, “Victor Babes” University of Medicine and Pharmacy, Timişoara, Romania; ^3^Nephrology Clinic – County Emergency Hospital “Pius Brinzeu”, Timişoara, Romania; ^4^Department of Functional Sciences, Center for Modeling Biological Systems and Data Analysis, “Victor Babes” University of Medicine and Pharmacy, Timişoara, Romania; ^5^Department of Gastroenterology and Hepatology, “Victor Babes” University of Medicine and Pharmacy, Timişoara, Romania; ^6^Department of Internal Medicine II – Division of Diabetes and Nutritional Disease, “Victor Babes” University of Medicine and Pharmacy, Timişoara, Romania; ^7^Diabetes and Nutritional Disease Clinic – County Emergency Hospital “Pius Brinzeu”, Timişoara, Romania; ^8^Department of Internal Medicine II – Division of Gastroenterology and Hepatology, Advanced Regional Research Center in Gastroenterology and Hepatology, “Victor Babes” University of Medicine and Pharmacy, Timişoara, Romania

**Keywords:** MAFLD, NAFLD, chronic kidney disease, transient elastography, controlled attenuation parameter

## Abstract

**Background:** Changing the term/concept of the non-alcoholic fatty liver disease (NAFLD) to metabolic dysfunction associated fatty liver disease (MAFLD) may broaden the pathological definition that can include chronic renal involvement, and, possibly, changes chronic kidney disease's (CKD's) epidemiological association with liver disease, because CKD is associated with metabolic disorders and almost all patients with CKD present some form of an atherogenic dyslipidemia. Our study explores the relationship between MAFLD and CKD using Transient Elastography (TE) with a Controlled Attenuated Parameter (CAP).

**Methods:** We evaluated 335 patients with diabetes with MAFLD and with high CKD risk using TE with CAP (FibroScan®). The CKD was defined according to Kidney Disease Improving Global Outcomes (KDIGO) 2012 guidelines. Logistic regression and stepwise multiple logistic regression were used to evaluate the factors associated with CKD. In addition, a receiver operating characteristic curve (ROC) analysis was used to assess the performance of CAP and TE in predicting CKD and its optimal threshold.

**Results:** The prevalence of CKD in our group was 60.8%. Patients with CKD had higher mean liver stiffness measurements (LSM) and CAP values than those without CKD. We found that hepatic steatosis was a better predictor of CKD than fibrosis. Univariate regression showed that CAP values >353 dB/m were predictive of CKD; while the multivariate regression analysis (after adjustment according to sex, body mass index (BMI), low-density lipoprotein cholesterol (LDLc), and high-density lipoprotein cholesterol (HDLc), and fasting glucose) showed that CAP values >353 dB/m were more strongly associated with the presence of CKD compared to the LSM (fibrosis) values.

**Conclusion:** In patients with MAFLD, CAP-assessed steatosis appears to be a better predictor of CKD compared to LSM-assessed hepatic fibrosis.

## Introduction

Recent data have proven that fatty liver, associated liver inflammation, and fibrosis [NAFLD and non-alcoholic steatohepatitis (NASH)] increase the risk of chronic kidney disease (CKD) (1). However, the NAFLD diagnosis does not include other liver disfunction/diseases (viral and toxic) associated with fatty liver. Therefore, the term MAFLD (metabolic dysfunction associated fatty liver disease) was recently proposed, which includes in its definition other liver diseases that are also associated with fatty liver. The definition of metabolic dysfunction associated fatty liver disease (MAFLD) is based on the evidence of hepatic steatosis, and the coexistence of overweight/obesity, or type 2 diabetes mellitus or the coexistence of two other risk factors related to metabolic dysregulation (waist circumference ≥102/88 cm in white men/women, low HDL-cholesterol, increase in serum triglyceride levels > 150 mg/dl, blood pressure >130/85, prediabetes, plasma C-reactive protein (CRP) >2.5, homeostatic model assessment (HOMA) score >2.5) (2). MAFLD can be diagnosed regardless of the daily alcohol consumption and other concomitant liver diseases. Furthermore, the relation between NAFLD/NASH and CKD has been explored, and the data have been extensively published (3). However, less is known about the relation between MAFLD and the risk of CKD. Our paper aimed to explore this relation using liver steatosis and liver fibrosis assessments by transient elastography (TE) with controlled attenuation parameter (CAP).

## Materials and Methods

### Patients

Patients who were previously diagnosed with MAFLD, by imaging methods (MRI or/and CAP), were prospectively enrolled in the study conducted in the Departments of Nephrology, Gastroenterology and Hepatology, and Diabetes and Metabolic Diseases in Timisoara Emergency County Hospital for a period of 1 year (January 2018 to December 2018). All patients were Caucasians and underwent transient elastography with controlled attenuation parameters. As inclusion criteria, patients were required to be over 18 years of age and with presence of MAFLD.

Chronic kidney disease (CKD) was defined by an albumin-to-creatinine ratio (A/Cr) >30 mg/g and/or estimated glomerular filtration rate (eGFR) <60 mL/min/1.73 m^2^ if persistent for more than 3 months. The eGFR was estimated using the CKD-Epi formula (4).

Exclusion criteria were as follows: pregnancy, ascites, outliers (subjects with inexplicable laboratory data values), decompensated liver disease, cardiac pacemaker, malignancy, end-stage renal disease, heart failure, unreliable or invalid TE and CAP measurements, and elevated aspartate aminotransferase (AST) and alanine aminotransferase (ALT) levels, which are more than five times the upper limit of normal. Known severe chronic liver disease- patients with liver cirrhosis (hepatitis B virus, hepatitis C virus, autoimmune hepatitis, or alcohol related liver disease) were also excluded from the study due to their previously established diagnostic and well-known etiology to avoid biases. The study protocol was conducted according to Declaration of Helsinki after the approval of “Pius Branzeu” County Emergency Clinical Hospital Ethical Committee (no. 131/25.10.2017). All patients gave their informed consent for the procedures.

### Clinical Assessments

Age, body mass index (BMI), waist circumference, and medical history were collected. Laboratory data including serum creatinine, AST, ALT, platelets, glycemia, cholesterol, triglycerides, HDL cholesterol, LDL cholesterol, urine albumin, and urine creatinine were also assessed in the same session with transient elastography, at 1, 2, and 3 months to identify the true CKD.

### Transient Elastography With Controlled Attenuation Parameter

Transient elastography was performed with a FibroScan® device (EchoSens, Paris, France). At the time of the procedure, patients were in fasting condition for more than 4 h. Patients were placed in a supine position, with their right arm in a maximum abduction, by intercostal approach, in the right liver lobe. In each patient, we aimed for 10 valid liver stiffness measurements (LSMs). The examination was performed using the M probe (standard probe, transducer frequency 3.5 MHz) or the XL probe (transducer frequency 2.5 MHz). The M and XL probes were used according to the European recommendation on M and XL probe selection (5). A median value of 10 valid LSMs was calculated, and the results were expressed in kilopascals (kPa). A reliable measurement was defined as the median value of 10 valid LSMs, with an interquartile range/median ratio (IQR/M) of < 30% (6).

To discriminate between fibrosis and steatosis stages, we used the TE and CAP cut-off values from a published multicentric trial compared with biopsy (TE: 8.2 kPa for F≥2, 9.7 kPa for F≥3, and 13.6 kPa for F = 4; CAP: 302 dB/m for S≥1, 331 dB/m for S≥2, and 337 dB/m for S3) (7).

### Statistical Analysis

MedCalc software (version 19.3.1) and Microsoft Excel 2019 were used for the statistical analysis. The distribution of numerical variables was tested with Kolmogorov–Smirnov test. In addition, we used *t-*test and ANOVA (for normal distributions), and Mann–Whitney *U*-test or Kruskal–Wallis tests (for non-normal distributions) to assess the differences between numerical variables. The proportions were analyzed using the chi-square test. Logistic regression and stepwise multiple logistic regression were used for the evaluation of the factors associated with CKD. The ROC analysis was used to assess the CAP and TE performance in predicting CKD and the optimal thresholds.

## Results

### Baseline Characteristics

**Figure 1:**
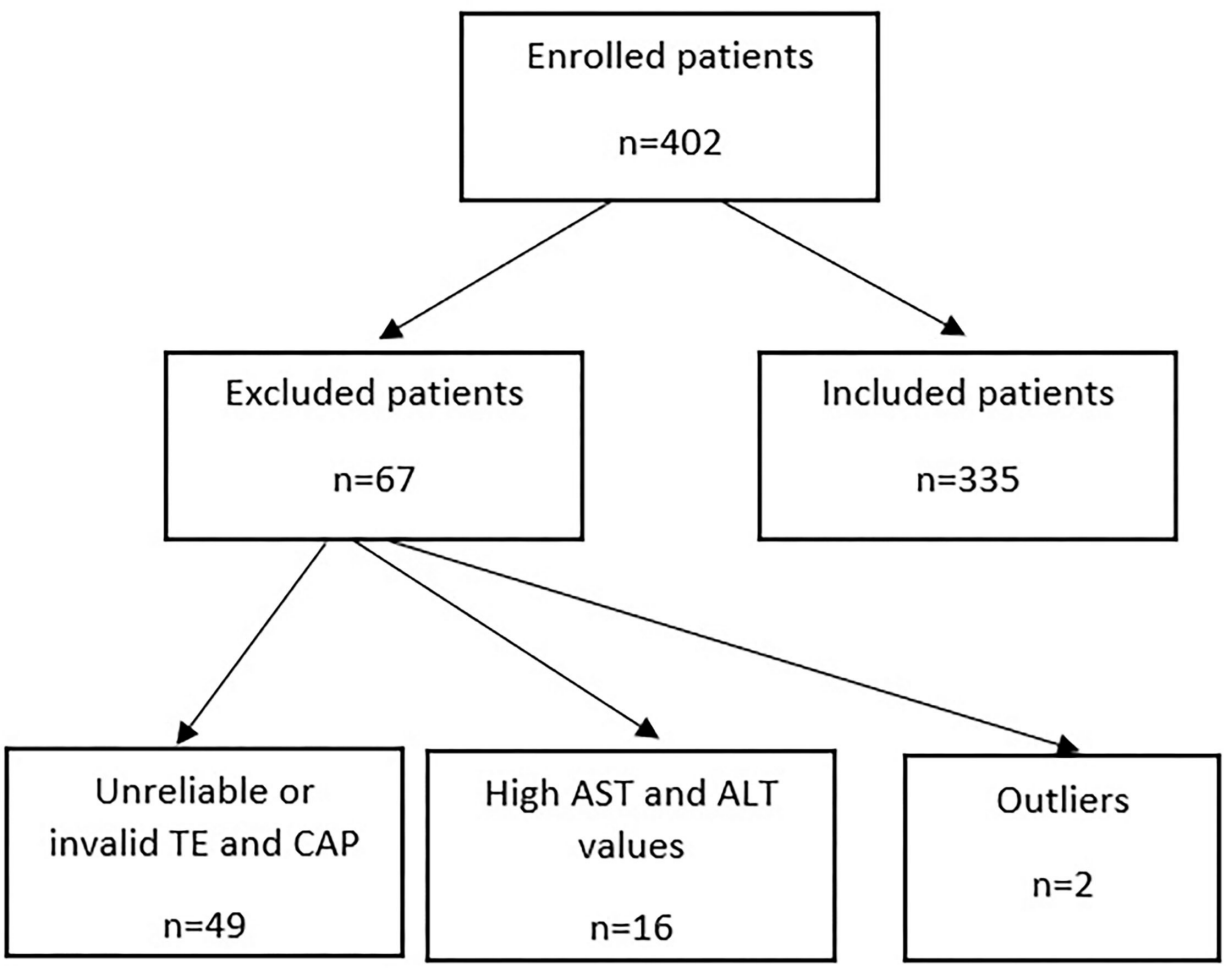
Study flowchart.

From the 402 patients, 355 patients met the inclusion criteria (Figure 1); of these, 44.1% were males. The average age was 60.84 ± 9.11 years. Among all patients, 60.8% had chronic kidney disease, and 83.80% had a history of hypertension. The characteristics of the patients are presented in Table 1.

**Table 1:** Baseline characteristics of MAFLD patients.

**Parameter**	***n* = 335**
Age (years)	60.84 ± 9.11
Gender (% male)	148 (44.1%)
BMI (kg/m^2^)	31.44 ± 5.98
Waist circumference (cm)	107.62 ± 14.65
AST (IU/L)	25.00 (9–159)
ALT (IU/L)	36.00 (9–200)
Platelets × 10^3^/mm^3^	243.52 ± 73.38
Total cholesterol (mg/dL)	189.22 ± 64.76
Triglycerides (mg/dL)	150 (30–420)
LDLc (mg/dL)	109.94 ± 40.09
HDLc (mg/dL)	43.16 ± 15.67
eGFR (mL/min/1.73m^2^)	71.19 ± 24.03
eGFR < 60 (mL/min/1.73m^2^)	193 (54.3%)
A/Cr > 30	178 (53.4%)
Creatinine (mg/dL)	1.09 ± 0.48
Fasting glucose (mg/dL)	180.38 ± 60.63
HbA1c (%)	8.53 ± 1.80
LSM (kPa)	8.03 ± 6.57
CAP (dB/m)	311.69 ± 59.94
Hypertension	281 (83.8%)
Dyslipidemia	125 (40.2%)
Fibrate treatment	108 (32.2%)
ACE inhibitors	97 (28.9%)
Diabetes duration	10.50 ± 8.51
Steatosis	257 (76.7%)
Severe steatosis	195 (58.2%)
**Fibrosis stages**
F0–1 F2 F3 F4	239 (71.4%) 60 (17.9%) 8 (2.3%) 28 (8.4%)
Significant fibrosis (>F2)	96 (28.6%)
Advanced fibrosis (>F3)	36 (10.7%)

*BMI, body mass index; ALT, alanine aminotransferase; AST, aspartate aminotransferase; eGFR, estimated glomerular filtration rate; HbA1c, hemoglobin A1c; HDLc, high-density lipoprotein cholesterol; LDLc, low-density lipoprotein cholesterol; LSM, liver stiffness measurement; CAP, controlled attenuation parameter; ACE, angiotensin converting enzyme*.

When comparing the two subgroups, with CKD and without CKD, subjects with CKD were older (age, *p* < 0.0001); more likely to be men (*p* < 0.0001); and had higher values of triglycerides (*p* = 0.04), fasting glucose, HbA1c *(p* = 0.01, *p* = 0.0008, respectively), mean LSM values (*p* = 0.04), mean at CAP (*p* = 0.03), and similar rates of hypertension, steatosis, and significant fibrosis (*p* > 0.05) (Table 2).

**Table 2:** Comparison between chronic kidney disease and non-chronic kidney disease groups.

**Parameter**	**CKD (*n* = 204)**	**No CKD (*n* = 131)**	***p*-value**
Age, years	62.64 ± 8.82	60.03 ± 6.88	0.008
Gender (% male)	91 (61.4%)	57 (38.6%)	0.0001
BMI (kg/m^2^)	31.35 ± 5.76	31.59 ± 6.32	0.72
Waist circumference (cm)	107.15± 15.49	108.32 ± 13.34	0.47
AST (IU/L)	24.50 (9–159)	23 (10–108)	0.05
ALT (IU/L)	41.12 (9–200)	37.00 (13–189)	0.86
Platelets × 10^3^/mm^3^	247.32 ± 81.01	237.67 ± 59.39	0.24
Total cholesterol (mg/dL)	191.21 ± 72.65	185.93 ± 49.27	0.46
Triglycerides (mg/dL)	157.5 (30–420)	141 (57–356)	0.04
LDLc (mg/dL)	109.34 ± 41.95	110.85 ± 37.20	0.73
HDLc (mg/dL)	41.00 ± 16.5	42.20 ± 14.19	0.49
eGFR (mL/min)	62.26 ± 24.68	85.21 ± 14.47	<0.0001
Creatinine (mg/dL)	1.23 ± 0.56	0.85 ± 0.16	<0.0001
Fasting glucose (mg/dL)	187.00 ± 62.95	170.14 ± 55.54	0.01
HbA1c (%)	8.79 ± 1.79	8.12 ± 1.74	0.0008
LSM (kPa)	8.64 ± 4.30	7.44 ± 3.15	0.04
CAP (dB/m)	320.09 ± 57.12	306.29 ± 61.21	0.03
Diabetes duration	11.10 ± 8.76	9.59 ± 7.51	0.17
Hypertension	174 (85.2%)	107 (81.6%)	0.38
Steatosis	150 (73.5%)	107 (81.6%)	0.008
Severe steatosis	113 (55.3%)	82 (62.5%)	0.19
Significant fibrosis	59 (28.9%)	37 (28.2%)	0.89
Advanced fibrosis	22 (10.7%)	14 (10.6%)	0.97
Dyslipidemia	69 (33.8%)	56 (42.7%)	0.10
Fibrate treatment	60 (29.4%)	48 (36.6%)	0.16
ACE inhibitors	21 (10.2%)	76 (58.0%)	<0.0001

*BMI, body mass index; ALT, alanine aminotransferase; AST, aspartate aminotransferase; CKD, chronic kidney disease; eGFR, estimated glomerular filtration rate; HbA1c, hemoglobin A1c; HDLc, high-density lipoprotein cholesterol; LDLc, low-density lipoprotein cholesterol; LSM, liver stiffness measurement; CAP, controlled attenuation parameter; ACE, angiotensin converting enzyme*.

No significant differences between the two subgroups were found regarding the waist circumference, BMI, AST, ALT, cholesterol, platelets, LDL, or HDL (Table 2).

Mean fibrosis LSM and CAP values were significantly higher in patients with CKD than in those without (8.64 ± 4.30 vs. 8.03 ± 6.57, *p* = 0.04; and 320.09 ± 57.12 vs. 306.29 ± 61.21, *p* = 0.04, respectively).

**Figure 2:**
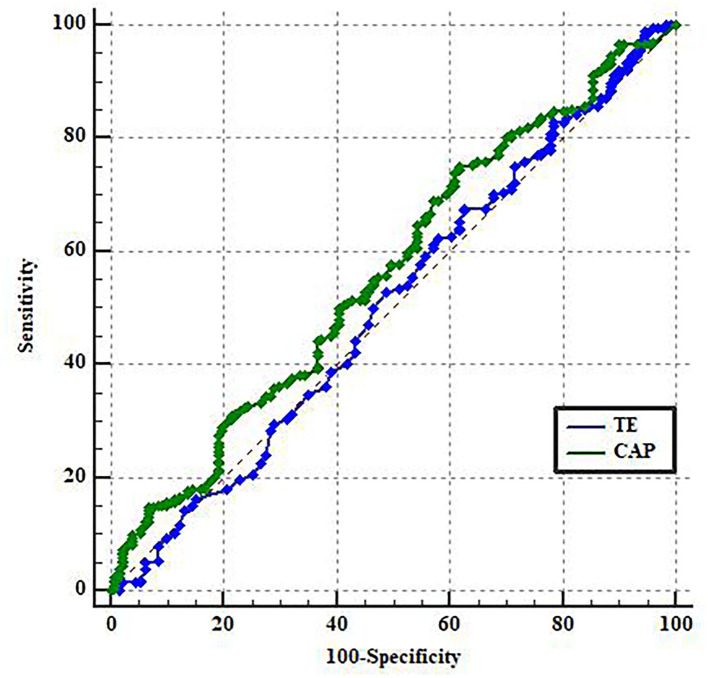
ROC curve for predicting CKD.

The ROC curves were used to determine if the transient elastography with controlled attenuation parameter could predict the presence of chronic kidney disease by assessing liver stiffness measurements or liver steatosis. The area under the receiver's operating characteristic curve of CAP was higher (AUC = 0.60, *p* = 0.01) than in TE (AUC = 0.51, *p* = 0.98), *p* = 0.001. The best CAP cut-off value was 353 dB/m, with a sensitivity of 75% and a specificity of 45.1% (Figure 2). A CAP value over 353 dB/m was strongly correlated with the presence of CKD (*r* = 0.89, *p* < 0.0001); The correlation between TE and the presence of CKD was *r* = 0.52, *p* = 0.12.

### Univariate and Multivariate Regression Analysis of Factors Involved in CKD Prediction

Six variables showed significant associations with CKD in the univariate analysis, including age (*p* < 0.0001), male gender (*p* < 0.0001), HbA1c (*p* = 0.002), fasting glucose (*p* = 0.04), CAP values (*p* = 0.03), angiotensin converting enzyme (ACE) inhibitors (*p* = 0.04), and creatinine (*p* < 0.001) (Table 3).

**Table 3:** Univariate and multivariate logistic regression analysis of factors associated with CKD.

**Factor**	**Univariate analysis**			**Multivariate analysis**		
	**OR**	**95% CI**	***p*-value**	**OR**	**95% CI**	***p*-value**
Age	1.08	1.00–1.20	<0.0001	1.04	1.00–1.07	0.04
Male	1.10	0.89–1.15	<0.0001	1.08	0.98–1.09	0.93
BMI	1.02	0.99–1.02	0.72	1.00	0.46–2.50	0.70
Triglycerides	1.00	0.98–1.00	0.44	0.99	0.99–1.00	0.46
LDLc	0.99	0.99–1.00	0.76	0.99	0.96–1.01	0.53
HDLc	0.99	0.99–1.01	0.43	1.01	0.98–1.04	0.33
HbA1c	1.01	1.00–1.10	0.002	1.00	1.00–1.01	0.02
Fasting glucose	1.00	0.99–1.05	0.04	1.00	0.99–1.01	0.11
Cholesterol	1.00	0.98–1.01	0.48	1.00	1.00–1.03	0.45
CAP	1.05	1.00–1.23	0.03	1.07	1.00–1.20	0.01
Creatinine	1.20	1.00–1.35	<0.0001	1.15	0.85–1.56	0.12
ALT	0.99	0.99–1.00	0.05	0.99	0.98–1.00	0.86
AST	0.99	0.97–1.00	0.38	1.00	0.98–1.02	0.67
TE	1.00	0.54–1.35	0.17	1.03	0.40–1.22	0.61
Severe steatosis	1.10	1.00–1.15	0.03	1.29	0.52–1.80	0.65
Advanced fibrosis	1.25	0.78–1.45	0.74	1.32	0.34–1.40	0.61
Hypertension	1.20	0.98–1.25	0.32	1.50	0.52–3.18	0.30
Dyslipidemia	1.00	0.99–1.02	0.59	1.10	0.50–2.39	0.75
Fibrate treatment	1.00	0.99–1.00	0.84	1.04	0.89–1.10	0.79
Duration of diabetes	0.99	0.97–1.00	0.16	0.99	0.95–1.03	0.97
ACE inhibitors	1.00	0.96–1.03	0.04	0.99	0.98–1.00	0.06

*BMI, body mass index; HbA1c, hemoglobin A1c; HDLc, high-density lipoprotein cholesterol; LDLc, low-density lipoprotein cholesterol; CAP, controlled attenuation parameter; ALT, alanine aminotransferase; AST, aspartate aminotransferase; LSM, liver stiffness measurements; OR, odds ratio; CI, confidence interval; ACE, angiotensin converting enzyme*.

In multiple logistic regression analysis, all variables showed in Table 3 were included, and after the adjustment of gender, fasting glucose, BMI, LDLc, and HDLc, CAP value, age, and HbA1c remained associated with the presence of CKD (Table 3).

## Discussion

For more than 35 years, the term NAFLD has covered conditions associated with the hepatic cell fat accumulation in the absence of evident causes like excess alcohol intake, viral hepatitis, drugs, inherited or acquired metabolic disorders, etc. NAFLD has two subtypes: the less severe non-alcoholic fatty liver (steatosis in more than 5% of hepatocytes without inflammation, necrosis, and fibrosis), and the more severe NASH characterized by steatosis, inflammation (lobular and portal), liver cell injury with the possibility of progression to fibrosis, cirrhosis, and end-stage liver disease. Some consider NAFLD a hepatic manifestation of metabolic syndrome (8). In the last decade, based mainly on observational studies, NAFLD was associated with an increased risk of cardiovascular disease and cardiovascular mortality. Therefore, it has been proven that the higher the severity of NAFLD, the higher the risk of fatal and non-fatal cardiovascular events (9). Considering the multiple common risk factors in observational studies and in two meta-analyses of those studies, NAFLD was also associated with both incident and prevalent chronic kidney disease. Additionally, it was found that the risk of CKD increased with the severity of the NAFLD (2, 10). The prevalence of CKD in cross-sectional studies varied between 5–47 and 2–29% in the longitudinal studies analyzed by Musso et al., depending on the definition of CKD used by the authors. The prevalence and incidence of CKD were significantly higher in NASH patients (11). A later meta-analysis by Mantovani et al. exploring the risk of incident CKD showed similar results (2). In both studies, both incident and prevalent CKD risk were significantly associated with the severity of liver fibrosis (2). Another studies by Ciardullo et al. (12, 13), Lomonaco et al. (14), and Yeung et al. (15) sustained the same, that in patients with NAFLD, liver fibrosis is associated with CKD and their prevalence of liver steatosis and liver fibrosis is greater in our study, but it may be due to the differences between the study cohorts. Our cohort is based only by participants who are Caucasian with type 2 diabetes, while the studies by Ciardullo et al. (12, 13) and Lomonaco et al. (14) are based on a mixed reced-ethnicity from United States, while the study conducted by Yeung et al. (15) is based entirely on Asian subjects. In recent years, it became evident that the excess accumulation of fat in liver cells may occur in many pathologic events beyond alcohol consumption, hepatitis B and C, autoimmune liver damage, and drugs. Additionally, the proven threshold for liver-safe alcohol consumption is not very clear (11). It has been suggested that even the alcohol-producing gut microbiota (*Klebsiella pneumoniae*) may influence the evolution of fatty liver disease (16). It seems that metabolic disorder is constantly associated with a fatty overload of the liver (17). The increasing number of patients with fatty liver; the possibility of progression to inflammation, fibrosis, cirrhosis, and complications as hepatic carcinoma, and the need for effective etiology-related interventions lead to a need for changing the nomenclature (paradigm?) of this disease. Thus, the term metabolic (disorder) associated fatty liver disease (MAFLD) was proposed (3). The definition of MAFLD requires the presence of hepatic steatosis associated with overweight/obesity (BMI >25 kg/m^2^ in white and >23 kg/m^2^ in Asian individuals), diabetes, or the presence of metabolic dysregulation. To establish metabolic dysfunction, at least two of the following characteristics must be present: waist circumference ≥102/88 cm in white men/women or ≥90/80 cm in Asian men/women, prediabetes, high serum C-reactive protein values, increased BP values or BP under treatment, low HDL-cholesterol, increase in serum triglyceride levels, and a HOMA score of >2.5. Specific individual characteristics such as genetic predisposition, age, sex, ethnicity, diet, metabolic status, and gut microbiota increase the risk of developing MAFLD. More importantly, MAFLD does not exclude other causes of fatty liver (17). The change of name and definition was associated with some changes in epidemiology. Though prevalence did not change significantly, the incidence of MAFLD decreased (by 25%), and it seems that hepatic fatty overload not associated with MAFLD definition criteria is less likely to develop into a significant liver disease (18). Moreover, it seems that the MAFLD criteria identify significant liver fibrosis better when compared to NAFLD (19).

Concerning the risk of CKD in patients with MAFLD, the data are scarce and contradictory. However, in analyzing the NHANES III 1988–94 database, the authors found that MAFLD can identify the patients with CKD risk better and that the risk of CKD and albuminuria is strongly correlated with the severity of liver fibrosis (the prevalence of CKD in MAFLD was 36.2%, and it was 30.2% in NAFLD) (20). However, the cross-sectional NHANES 2017–2018 study did not confirm these results (21).

Our study investigated patients with established MAFLD and its association with CKD (multiple risk factors: DM all cases, poor DM control, HT in 83.8%, average BMI 31.44 ± 5.98 kg/m^2^, and hypercholesterolemia). Under these conditions, the prevalence of CKD was high (60.8%). Subjects with CKD, who were older than those without, were more likely to be men, and had higher triglyceride values, poorer glycemic control, and higher rates of hypertension (as expected). Concerning the TE findings with CAP, higher mean LSM values and higher mean CAP were recorded in patients with CKD, and similar rates of steatosis and fibrosis were evidenced in patients with patients (Table 2). When ROC curves were used to determine the presence of CKD related to liver fibrosis and steatosis, steatosis showed a higher AUC than fibrosis. Univariate regression analysis showed that the severe steatosis and CAP values (along with age, male gender, HbA1c values, and fasting glucose values), but not severe fibrosis and LSM values, were associated with CKD. The stepwise multiple logistic regression analysis confirmed our hypothesis; that is, after adjustment for gender, fasting glucose, BMI, LDLc, and HDLc, CAP values higher than 353 dB/m were strongly correlated with the presence of CKD (other predictors were age and HbA1C), and not with fibrosis or LSM values.

We explored some regression models for CKD prediction in combination with CAP values to exclude bias factors that may be involved in CKD (i.e., hypertension, dyslipidemia, fibrate treatment, and duration of diabetes). In all models, the higher CAP values were independently associated with CKD. Although all patients with CKD tend to develop dyslipidemia (more frequently the very atherogenic type) (22), the question of whether CKD may influence steatosis in patients with MAFLD patients remains unanswered. Our study has some limitations: All patients were type two diabetes with steatosis; the lack of a control group to sustain the findings; and the fact that the study was a cross-sectional one.

## Conclusions

In patients with established MAFLD and with multiple metabolic risk factors for CKD, the liver fatty overload evaluated with CAP seems to be a better predictor of CKD than LSM and fibrosis. However, more studies with a higher number of patients are needed to confirm our results. Furthermore, some questions remain to be answered in future research: In the liver–kidney crosstalk, how is CKD influencing the MAFLD outcomes, since CKD is a one-way pathological process? Additionally, does MAFLD influence CKD progression to an end-stage kidney disease.

## Data Availability Statement

The raw data supporting the conclusions of this article will be made available by the authors, without undue reservation.

## Ethics Statement

The studies involving human participants were reviewed and approved by County Emergency Hospital “Pius Branzeu” Timisoara Ethical Committee. The patients/participants provided their written informed consent to participate in this study.

## Author Contributions

LM and AS: conceptualization. AM and FB: methodology. RL and BT: software. AS, IS, and RT: validation. IG, FB, and FG: formal analysis. RL, LM, and LC: investigation. AS, RT, and IS: resources. LM, RL, and AM: data curation. LM, RL, and VT: writing—original draft preparation. AS, IG, AM, and FB: writing—review and editing. IG, AM, and NO: visualization. AS: supervision. All authors have read and agreed to the published version of the manuscript.

## Conflict of Interest

The authors declare that the research was conducted in the absence of any commercial or financial relationships that could be construed as a potential conflict of interest.

## Publisher's Note

All claims expressed in this article are solely those of the authors and do not necessarily represent those of their affiliated organizations, or those of the publisher, the editors and the reviewers. Any product that may be evaluated in this article, or claim that may be made by its manufacturer, is not guaranteed or endorsed by the publisher.
